# Quercetin and cancer: new insights into its therapeutic effects on ovarian cancer cells

**DOI:** 10.1186/s13578-020-00397-0

**Published:** 2020-03-10

**Authors:** Asma Vafadar, Zahra Shabaninejad, Ahmad Movahedpour, Farzaneh Fallahi, Mona Taghavipour, Younes Ghasemi, Maryam Akbari, Alimohammad Shafiee, Sarah Hajighadimi, Sanaz Moradizarmehri, Ebrahim Razi, Amir Savardashtaki, Hamed Mirzaei

**Affiliations:** 1 Department of Medical Biotechnology, School of Advanced Medical Sciences and Technologies, Shiraz University of Medical Sciences, Shiraz, Iran; 2 Department of Nanotechnology, Faculty of Biological Sciences, Tarbiat Modares University, Tehran, Iran; 3 Pharmaceutical Sciences Research Center, Shiraz University of Medical Sciences, Shiraz, Iran; 4 Student Research Committee, Shiraz University of Medical Sciences, Shiraz, Iran; 5 Research Center for Biochemistry and Nutrition in Metabolic Diseases, Institute for Basic Sciences, Kashan University of Medical Sciences, Kashan, I.R. of Iran; 6 Department of Gynecology and Obstetrics, Ramsar Campus, Mazandaran University of Medical Sciences, Sari, Iran; 7 Department of Pharmaceutical Biotechnology, School of Pharmacy and Pharmaceutical Sciences Research Center, Shiraz University of Medical Sciences, Shiraz, Iran; 8 Department of Surgery, Kashan University of Medical Sciences, Kashan, Iran; 9 Division of General Internal Medicine, Toronto General Hospital, Toronto, ON Canada; 10 The Advocate Center for Clinical Research, Ayatollah Yasrebi Hospital, Kashan, Iran

**Keywords:** Quercetin, Ovarian cancer, Therapy

## Abstract

Ovarian cancer is known as a serious malignancy that affects women’s reproductive tract and can considerably threat their health. A wide range of molecular mechanisms and genetic modifications have been involved in ovarian cancer pathogenesis making it difficult to develop effective therapeutic platforms. Hence, discovery and developing new therapeutic approaches are required. Medicinal plants, as a new source of drugs, could potentially be used alone or in combination with other medicines in the treatment of various cancers such as ovarian cancer. Among various natural compounds, quercetin has shown great anti-cancer and anti-inflammatory properties. In vitro and in vivo experiments have revealed that quercetin possesses a cytotoxic impact on ovarian cancer cells. Despite obtaining good results both in vitro and in vivo, few clinical studies have assessed the anti-cancer effects of quercetin particularly in the ovarian cancer. Therefore, it seems that further clinical studies may introduce quercetin as therapeutic agent alone or in combination with other chemotherapy drugs to the clinical setting. Here, we not only summarize the anti-cancer effects of quercetin but also highlight the therapeutic effects of quercetin in the ovarian cancer.

## Introduction

According to a 2018 report, ovarian cancer ranked as the seventh most prevalent female cancer across the world, with approximately 240,000 new subjects [1]. Ovarian cancer usually is diagnosed in advanced stages, since it is associated with silent and unclear symptoms [2]. Despite extensive knowledge obtained on this complication in recent years, the rate of survival has not improved significantly because of some challenges in diagnosing and treatment it as early as possible [3]. Different modifications in molecular and genetic levels in ovarian cancer result in some challenges to establish a therapeutic platform [4].

According to a wide range of experiments, phytochemicals including polyphenols, flavones, as well as flavonoids possess considerable anti-cancer features, which can be employed against different kinds of cancers [5]. In this regard, quercetin, which can be extensively observed in daily foods including nuts, teas, vegetables, different plants, and in general the daily dietary program of people, is a common phytochemical [6]. Moreover, this supplementary agent is commercially accessible, while its oral application at a dose of 1 g per day is safe enough and can be absorbed up to 60% [7]. An extensive range of pharmacologic activities has been reported for quercetin, including anti-oxidant, anti-diabetes, anti-inflammation, as well as anti-proliferation [8, 9]. Quercetin, 2-(3,4-dihy-droxyphenyl)-3,5,7-trihydroxy4H-chromen-4-one includes two benzene rings named A and B, and joined through a 3-carbone heterocyclic pyrone one [9]. Since two antioxidant pharmacophores are present in the structure of quercetin, it can largely remove free radicals and join to transitional metal ions [9]. Moreover, catechol along with the OH group presenting at the position C3 in the structure of quercetin is an ideal arrangement to scavenge free radicals [9]. This agent is a pental-hydroxyl-flavonol consisting of 5 hydroxyl groups on the flavonol structure at 3, 30, 40 5, and 7 position carbons. Replacement of various functional groups leads to different biochemical as well as pharmacologic properties of quercetin [10].

Some studies have indicated that quercetin may exist in two states: (i) free or aglycone and (ii) mixed with different molecules. It can be interacted with molecules including, carbohydrates, lipids, alcohols, as well as sulfate group to generate derivatives of quercetin such as quercetin glycoside, prenylated quercetin, quercetin ethers and quercetin sulfate [10].

Furthermore, significant number of studies focused on anti-cancer properties of this bioactive compound. Several pathways have been identified which are affected by quercetin in different cancers [11, 12]. Based on available evidences, quercetin can inhibit a broad range of cancers such as breast [13], lung [14], nasopharyngeal [15], kidney [16], colorectal [17], prostate [18], pancreatic [19], as well as ovarian [20] cancers. According to the previous literature, consuming vegetables that are rich in quercetin has been associated with lower risks of ovarian cancer [21]. Similarly, consuming fruits with high levels of quercetin, including apples as well as citrus or their juice can decrease occurrence of ovarian cancer [22].

Quercetin is not harmful for healthy cells, while it can impose cytotoxic effects on cancer cells through several mechanisms, making it a good candidate to treat ovarian cancer or to be employed as a supplementary factor along with other anti-cancer medications [23]. In this review, we summarized the recent and eminent researches on quercetin properties in cancer therapy, especially ovarian cancer.

## Quercetin and its biologic functions

**Fig. 1:**
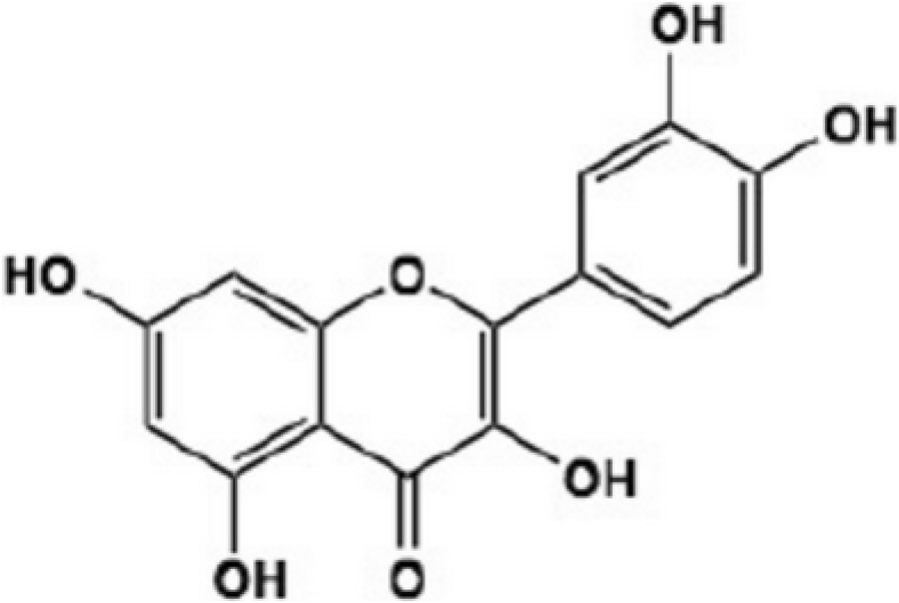
Chemical structure of quercetin (Q1)

Quercetin is an ordinary flavonoid that is pervasive in various types of foods and plant. Quercetin glycosides are the dominant flavonoid content that can be found in propolis along with other healthy foods, including fruits and vegetables; particularly onion, broccoli, apple, tea, as well as red wine. International Union of Pure and Applied Chemistry (IUPAC) has called it 3,3′,4′,5,7-pentahydroxyflavone (Fig. 1). Another name for this agent is 3,3′,4′,5,7-pentahydroxy-2-phenylchromen-4-one, and five hydroxyl groups are present at the positions 3, 5, 7, 3′ and 4′ of the flavonoid as its typical characteristic [24–26]. Quercetin can be used as a nutritional supplement. Quercetin is reported to have several beneficial effects on human health such as anti-inflammatory effects, cardiovascular protection and anticancer activity. It can act as an anti-cancer, anti-tumor, anti-ulcer, anti-allergy, anti-viral, anti-inflammation, and anti-diabetes agent, exerting gastro-protection, anti-hypertension, immune-modulation, as well as anti-infection features are among its advantageous effects [27].

Ferry and colleagues have investigated the pharmacokinetic effects of intravenously injected quercetin in the patients with cancer at doses of 60–2000 mg/m^2^. The safe dose of 945 mg/m^2^ was identified by the researchers. At higher doses that were toxic, vomiting, high blood pressure, nephrotoxicity, as well as decreased serum potassium could be observed. The dispensation and removal half-life of quercetin applied intravenously, is 0.7–7.8 min, and 3.8–86 min, correspondingly. Elimination is 0.84 L/min/m^2^, and dispensation content is 3.7 L/m^2^ [28]. Pharmacokinetic characteristics of oral administration of 8, 20, and 500 mg quercetin aglycone were examined in healthy volunteers by Erlund et al. [29]. On the other hand, Graefe and colleagues examined the pharmacokinetic features of this agent at a dose of 200 mg. C_max_ as well as T_max_ of quercetin have been reported to be 2.3 ± 1.5 µg/mL and 0.7 ± 0.3 h, correspondingly [30].

**Fig. 2:**
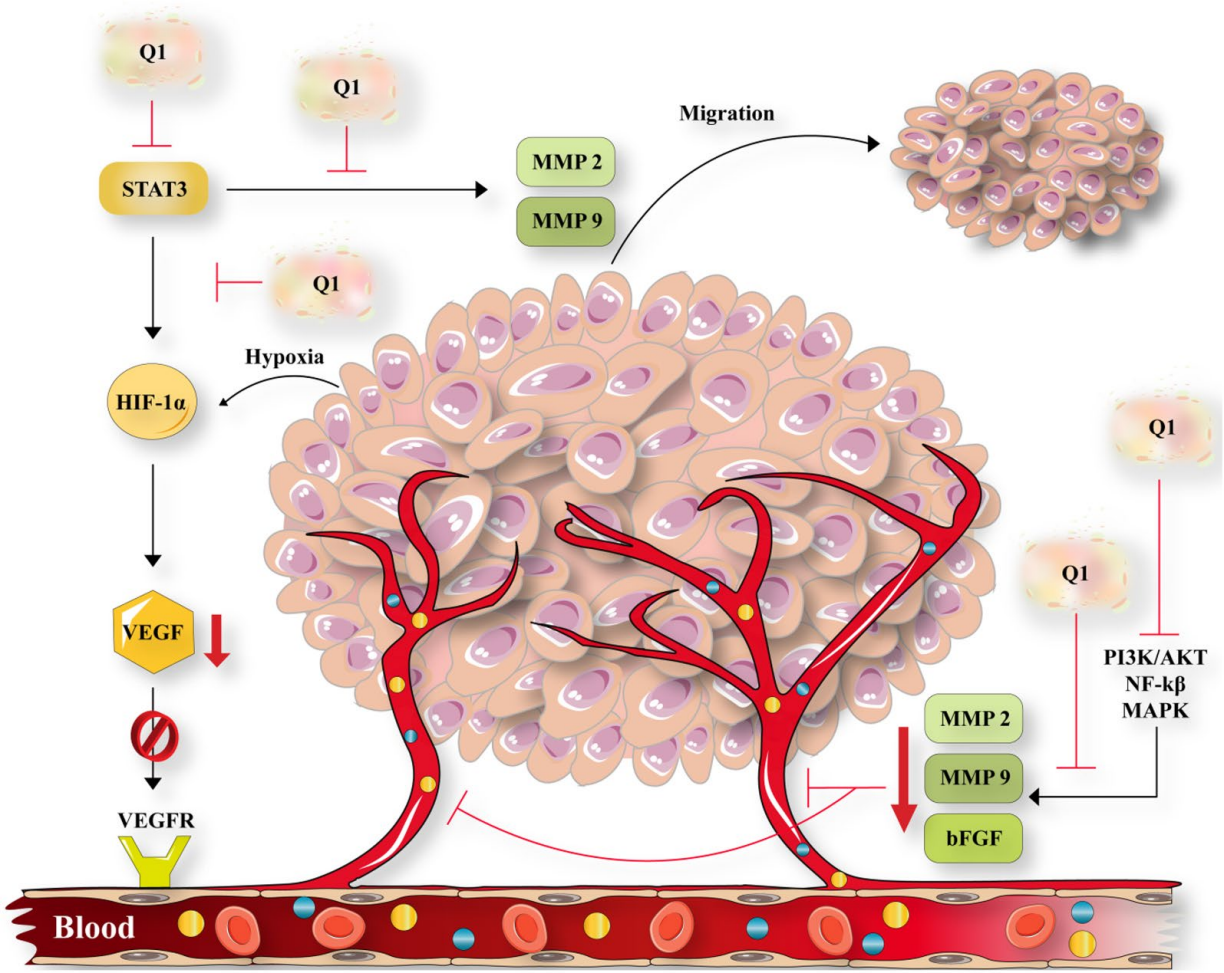
Anti-angiogenesis effects of quercetin

Angiogenesis is one of very important cancer-related processes. It has been showed, quercetin exerts its anti-angiogenesis effects in various cancers (Fig. 2) [31]. Moreover, quercetin is capable to protect against free radicals including smoking. Free radicals originated from cigarette tar can impose irreparable damages to erythrocyte membranes. In addition, quercetin and its conjugate metabolites have been reported to possess the potential to protect erythrocytes against damage of the membrane resulted from smoking [32].

Inflammation, through which self-protecting measures are taken, is a type of biologic reaction in the case human body experiences damaging or troublesome stimuli. The aim of this process is removing of the injured cells, pathogens, or other adverse stimuli, while beginning the treatment procedure. Inflammatory conditions are not essentially equal to infection. Infection is usually resulted from a viral, bacterial, or fungal source, but inflammatory processes include the reaction of the human body toward healing itself [33, 34]. The potential of quercetin in modulation of inflammation is one of its critical and considerable features. Accordingly, inflammatory enzymes of cyclooxygenase (COX) and lipoxygenase are inhibited by this agent and consequently inflammatory mediators including prostaglandins as well as leukotrienes are reduced [33, 34].

Nutritionists at Michigan State University studied the general effects of dietary flavonoids including quercetin as systemic anti-inflammation elements [35]. Increased level of C-reactive protein (CRP) was related to several disease conditions consisting of being obese, experiencing heart problems, as well as lupus. The researchers concluded that consumption of specific foods lowers the levels of inflammation risk factors (CRP).

Pre-clinical investigations have shown that quercetin can significantly reduce the levels of inflammation moderators including NO synthase, COX-2, as well as CRP in human hepatocyte-derived cell line [36]. Regarding experiments carried out on rats, quercetin (80 mg equivalent dose) hindered acute and chronic inflammatory conditions, while it also indicated considerable anti-arthritic properties against adjuvant-induced arthritis [37, 38].

In a study performed by Askari and colleagues, the impact of 2-month flavonoid quercetin (500 mg) supplement was examined in healthy amateur sportsmen having regular exercise. The results of this study indicated a considerable reduction in CRP levels [39]. Nevertheless, in pathologic conditions, this agent did not cause considerable changes in CRP levels in women who had rheumatoid arthritis (RA). The study continued in an 8-week interval, and the RA patients received 500 mg/day of quercetin [40]. Moreover, quercetin hampers the accumulation of uric acid due to its capability of inhibiting xanthine oxidase, which is probably beneficial in patients suffering gout [41].

Quercetin has also antibacterial potential regarding approximately all kinds of bacteria, and especially influences gastrointestinal, respiratory, urinary, as well as dermal systems. Its anti-infection and anti-replication properties are probably due to its antiviral features. Viruses that usually react to flavonoids include adenovirus, herpes simplex virus, Japanese encephalitis virus, as well as respiratory syncytial virus [42, 43].

Quercetin possesses anti-allergic properties through inhibition of releasing histamine from mast cells along with other allergic substances; therefore, it plays the role of a natural antihistamine. The potential of quercetin in prevention of allergies results in significant implications to employ it to treat and prevent asthma and bronchitis. The cell membranes of mast cells have the role of an immune gateway for the brain, the environment, as well as emotional stress [44].

Although a broad range of biologic advantages was mentioned above, quercetin pharmaceutical application and clinical conditions have been constrained due to its low hydro-solubility, lack of stability in physiologic conditions along with low bioavailability. [45]. It is worth considering that the anticancer properties of quercetin may be dependent to some extent on its metabolites [46–48]. Currently, several analogs having better solubility and possessing various biologic features such as biphasic, inotropic and lusitropic impacts have been introduced [49, 50].

A review described the anticancer properties of a small collection of quercetin analogs, while the hydrophilic-free OH have been completely or partially covered with groups that are conveniently removed considering in vivo conditions. Particularly, hydroxyl groups of the natural compound were replaced with different combinations, including acetyl esters, ethyl or benzyl ethers, or diphenyl ketal of the catechol system. Alterations made in the chemicals resulted in compounds that had better anti-cancer properties regarding quercetin. In addition, the potential of the proposed analogs in inhibition of human topoisomerases I and II was examined through direct enzymatic assays along with docking analyses [51]. Eventually, findings regarding the capability of lead compounds in modulation of intracellular ROS generations could be recorded.

## The effect of quercetin on cancers

Despite many advances in treating cancer, it is still known as a life-threatening malignancy in human. Although chemotherapy is employed as the conventional treatment for cancer, it has been illustrated that its usage is restricted in most cancers because of reasons including chemotherapy resistance and side effects.

**Fig. 3:**
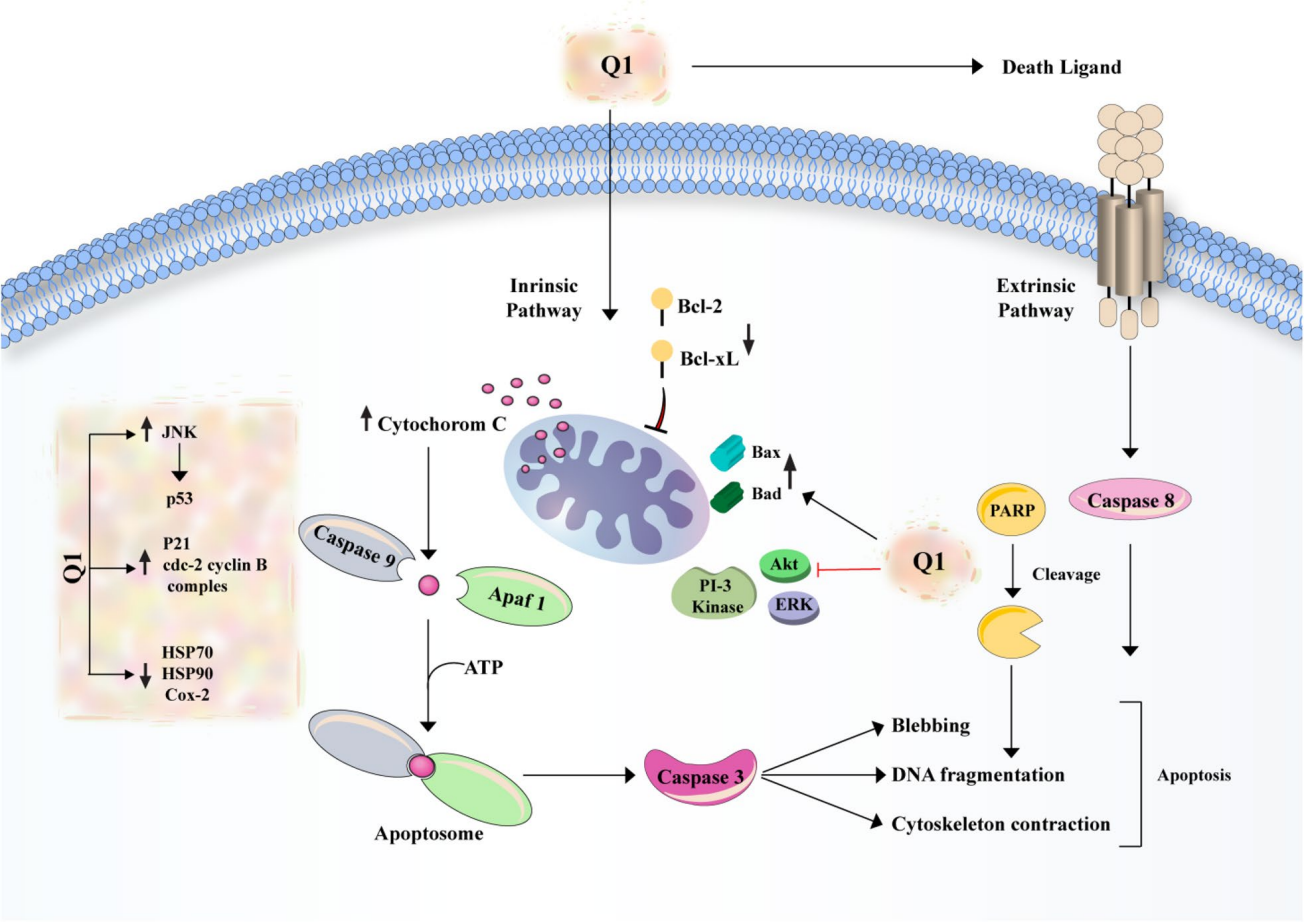
A schema of the effects of quercetin on apoptosis

Nowadays, natural compounds such as quercetin have been recognized as significant agent for preventing and healing cancer because of their predictable performance, high therapeutic potential and their low toxicity. It seems that quercetin plays an important role as an anti-proliferative and anticancer agent and also stimulates apoptosis (Fig. 3) [52]. Numerous studies probed the influence of isolated quercetin compound in different cancer cell lines. Obviously, quercetin has repressed the proliferation of cancers such as gastric cancer (GC) [53], breast cancer [54], colorectal cancer (CRC) [55], oral cancer [56], liver cancer [57], prostate cancer [58], thyroid cancer [59], leukemia [60], pancreatic cancer [61] and lung cancer [62].

### Quercetin and gastrointestinal cancers

Gastrointestinal cancers, the leading causes of cancer-related mortality worldwide, account for one fourth of all malignancies and for 9% of all cancer deaths [63], have elevating rate of incidence and heavy health burden on society [64]. Quercetin significantly suppressed GC cell viability, migration and invasion activities via decreasing expression of urokinase plasminogen activator (uPA) and uPA receptor (uPAR) proteins, which are strongly associated with GC metastasis. Quercetin could be used as an anti-metastatic agent against GC metastasis cells through interfering with uPA/uPAR systems, AMPKα, NF-κβ, ERK1/2, and PKC-δ regulation [65]. Recently, quercetin was carried with a novel phenyl boronic acid (PBA) conjugated ZnO nanoparticles (PBA-ZnO-Q) construct. It was determined that PBA-ZnO-Q can be efficient in diminishing tumor growth, in vivo. *KRAS* is a mutant oncogenic gene in up to 40% of CRC patient that has been confirmed leads to resistant chemotherapy and poor prognosis in CRC cases [17, 66, 67]. Investigation of quercetin effects on CRC cells carrying *KRAS* mutant gene revealed that quercetin could decrease the cell viability and increase apoptosis in cancer cells based on MTT assay and colony formation methods. The possible underlying mechanisms are the AKT pathway repression and activation of the c-Jun N-terminal kinase (JNK) pathway in KRAS-mutant cells. [17]. Gold nanoparticles (AuNPs)-quercetin into poly dl-lactide-*co*-glycolide nanoparticles significantly repressed the cell proliferation, colony formation, progression and cell migration of liver cancer. According to data, this construct containing quercetin increased the apoptosis via enhancing of caspase-3, caspase-9, and provoked more freeing of cytochrome c (cyto-c). Quercetin nanoparticle also repressed Akt/ERK1/2 signaling pathway, telomerase reverse transcriptase (hTERT) via impending AP-2β/hTERT, and cyclooxygenase 2(COX-2) through inactivated the NF-κB/COX-2. [68]. Treating HSC-6 and SCC-9 cell lies, oral cancer originated cell lines, with 50 µM of quercetin showed inhibited cell viability, migration, and invasion via attenuated the excesses of MMP-9 and MMP-2 in those cells. Beside, quercetin treatment alleviated the miR-16 expression, which were upregulated in oral cancer cell line and tissues.

### Quercetin and hematological cancers

Hematological cancers are diseases of abnormal progenitor and stem cells, originating from epigenetic and genetic alterations that lead to the dysregulation of differentiation, proliferation, and self-renewal of cells [69]. In the majority of these malignancies, the bone marrow, along with peripheral blood, lymphatic nodes, and spleen, as secondary lymphoid organs, are main locations for tumour localisation [70]. Quercetin has been also proved to possess beneficial effects on hematological cancers. He and his co-workers carried out an investigation and showed that quercetin inhibits proliferation of MM.1R, ARP-1, and RPMI8226 multiple myeloma cell lines through inducing apoptosis as well as cell cycle arrest in the G2/M phase. Moreover, the combination of quercetin with dexamethasone further enhanced apoptosis and inhibited tumor growth [71]. In another study, Ma et al. reported that quercetin inhibits multiple myeloma cells proliferation via down-regulating the expression of IQGAP1 and ERK activation [72]. It has been indicated that quercetin suppresses STAT3 and PI3K/AKT/mTOR pathways in primary effusion lymphoma (PEL) cells leading to downregulate the prosurvival cellular proteins expression, including cMyc, cyclin D1, and c-FLIP. Furthermore, quercetin decreased the IL-6 and IL-10 release, resulting in PEL cell death. A prosurvival autophagy was also mediated by quercetin, which promoted the cytotoxic effects of bortezomib, a proteasomal inhibitor [73]. Ha and colleagues demonstrated that quercetin induced cytoprotective autophagy and intrinsic apoptosis, and the suppression of autophagy with chloroquine potentiates apoptotic ability of quercetin in human T cell acute lymphoblastic leukemia Jurkat clones [74]. According to former studies, it was clarified TNF-related apoptosis-inducing ligand (TRAIL) as a biological cytokine playing important role in promoting apoptosis through attaching to its agonist receptors in cancer cells but its utilization has been limited because of resistance to some cancers [75, 76]. For this reason, a group of scientists has explored the synergist effect of quercetin and TRAIL in human myeloid leukemia KG-1 cells and showed that quercetin can be employed as a sensitizing factor alongside with TRAIL promoted the influence of TRAIL-induced apoptosis in KG-1 cells. On the basis of quantitative Real-time PCR results, it was observed the expression level of death receptor (DR) genes including DR4 and DR5 after treating with quercetin were increased and also the p65 expression and antiapoptotic proteins including c-IAP1, c-IAP2, and XIAP were reduced [75].

### Quercetin and gynecological cancers

Gynecologic malignancies are the fourth most prevalent type of cancers in females and influence the organs and tissue of the reproductive system of women, such as the vulva-vagina, cervix, uterus, and ovaries [77–79]. To prove the effectiveness of quercetin for the treatment of gynecological malignancies, numerous researches have been performed. For example, Lin et al. revealed that quercetin, by declining the expression of UBE2S, which is highly expressed in malignant cancers contributed to cell motility via epithelial–mesenchymal transition signaling, inhibits the invasion of cervical cancer cells [80].

### Other cancers

Tummala et al. showed that quercetin downregulated AR-V7 and hnRNPA1 expression, antagonized androgen receptor signaling, and promoted the sensitization of enzalutamide-resistant prostate cancer cells to enzalutamide treatment [81]. In the case of melanoma therapy, it was recently stated that quercetin inhibited the viability of A375SM melanoma cells through apoptosis induction. Additionally, it reduced the tumor volume in vivo and decreased the proliferation of cancerous cells in vitro [82]. Moreover, quercetin is a potent anticancer agent for the treatment of brain tumors, such as glioblastoma multiforme, as reviewed by Tavana et el. [83]. In human papillary thyroid cancer cell line (B-CPAP) quercetin significantly decreased cell proliferation and promoted apoptosis through caspase activation. It also provoked cell apoptosis through Hsp90 expression that may be associated with the reduction of chymotrypsin-like proteasome activity. The elimination of chymotrypsin-like proteasome activity represses growth and led to cell death in thyroid cancer cells [59]. Since low bioavailability of quercetin, Quagliariello et al. were used a hyaluronic acid (HA) hydrogel as a carrier of quercetin and have explored the result of drug treatment alone or in combination to SNS-314 that is an anti-Aurora kinase in human medullary and papillary thyroid cancer cells. It was noted that HA acted as a ligand of CD44 which is sufficiently increased in various cancer cells and related to tumor progression [84, 85]. On the other hand, incorporation of quercetin and SNS-314 caused a synergistic cytotoxic influence on both cell lines with an important reduction of the IC50 value [85]. Wiśniewska and colleagues have demonstrated the impact of quercetin on the essential cytoskeletal elements, such as microfilaments, microtubules, cytoskeleton-driven processes and vimentin intermediate filaments in A549 non-small cell lung cancer cells. Their results revealed that quercetin induced apoptosis via the regulation of BCL2/BAX, also necrosis and mitotic catastrophe, and repressed the migratory of those cells. The disassembling influence of quercetin on vimentin filaments, microtubules, and microfilaments accompanied by its suppression effect on N-cadherin and vimentin expression might be responsible for the reduced migration of A549 cells in reply to quercetin therapy. It is also reported that the probable mechanism underlying quercetin -induced mitotic disaster comprise the disturbance of mitotic microtubules results in monopolar spindle development, and subsequently the collapse of cytokinesis. They also proposed that quercetin can lead to the destruction of actin filaments leading to cytokinesis failure [86].

After 72 h treatment with 40 µM quercetin, decreased cell viability and promoted apoptosis follow by necrosis was reported in prostate cancer (Pca) cells treated compare to untreated ones. In addition, quercetin has impressing effects on the mitochondrial integrity and also depending on oxidation situation of the cells, can serve as an antioxidant or as a pro-oxidant for balancing reactive oxygen species (ROS) production in PCa cells. According to this study, quercetin repressed PCa via alleviating cell survival, frustrating anti-apoptotic pathways and also participate in MAPK, Akt, and NF-κB signaling pathways in different PCa cell lines with p53 mutated or without p53 mutated. Collectively, this research declared quercetin via regulation of ROS, Akt, and NF-κB pathways show an anti-cancer effect and could be utilized as a chemotherapeutic medicine to promote clinical results of PCa cases [87].

Nwaeburu et al. have surveyed the profiling of miRNA expression in AsPC1 (Pancreatic cancer cell line) cells before and after treatment with quercetin and indicated quercetin induced miR-200b-3p expression. This miRNA has a significant role in the choice of a Pancreatic Ductal Adenocarcinoma (PDA) cell to offer either symmetric or asymmetric division through regulating Notch signaling [88]. Symmetric division causes two equal daughter cells and leads to exponential tumor growth but asymmetric cell division produce two different daughter cells and is presented through cancer stem cells(CSCs) for homeostasis [89–91]. Also, it was proved that miR-200b expressed highly in the normal pancreas cell and of course down-regulated in PDA cells [88, 92]. After treatment of PDA cell with quercetin, the expression level of miR-200 was modulated which resulting to cell fate control and repression of cancer initiation, proliferation, invasion [88, 93, 94]. Table 1 listed various studies on the anti-cancer effects of quercetin.

**Table 1:** The therapeutic effects of quercetin on various cancers

Cancer	Type of quercetin	Mechanism(s) and Effect(s)	Dose(s)	Model	Refs.
Gastric cancer (GC)	Quercetin	Antimetastatic effects on GC Cells via interruption of uPA/uPAR Function by Modulating NF-κb, PKC-δ, ERK1/2, and AMPKα	10 μM	Human, in vitro	[65]
	Quercetin	Suppresses the growth of human GC stem cells by provoking mitochondrial-dependent apoptosis by the repression of PI3K/Akt signaling	20 µM	In vitro	[120]
	Q1 isolated from Polygonum capitatum (PC)	Modulation of apoptosis rate of GC cells via controlling the levels of p38MAPK, BCL-2 and BAX genes	10 μg/mL, 64 μg/mL	In vitro, in vivo	[121]
Breast cancer (BC)	Q1 targeted via phenyl boronic acid and zinc oxide nanoparticles (PBA-ZnO-Q)	Induced apoptotic cell death in BC cells via intensified combinatorial ROS(oxidative stress and mitochondrial damage)	8 μg/mL, 10 mg/kg	In vitro, in vivo	[122]
	Quercetin	Induced the expressions of Bax and cleaved caspase-3 and represses the proliferation and invasion activities by overexpression of miR-146a	80 µm/mL	In vitro, in vivo	[123]
	Quercetin	Represses BC stem cells (CD44+/CD24−) by restraining the PI3K/Akt/mTOR-signaling pathway	50 μM	In vitro, in vivo	[13]
Colorectal cancer (CRC)	Quercetin	Promoted apoptosis in KRAS-mutant CRC cells via activation of JNK signaling pathways and repression of the AKT pathway	100 µM	In vitro	[17]
	Quercetin	Promote 5-fluorouracil-induced apoptosis in MSI CRC cells via p53 regulation	1 µM, 100 µM	In vitro	[124]
	Quercetin	Provokes cell cycle arrest and apoptosis in stem cells of human colorectal HT29 cancer cell line, improves anticancer effects of doxorubicin	75 µM	In vitro	[125]
Oral cancer	Quercetin	Represses cell viability, migration and invasion through modulating miR-16/HOXA10 axis	50 μM	Human, in vitro	[126]
	Quercetin	Enhanced apoptosis in human oral cancer SAS cells by mitochondria and endoplasmic reticulum-mediated signaling pathways	40 µM	In vitro	[127]
	Quercetin	Decreases tumor rate and provokes cancer-cell apoptosis via regulation of NF-κB signaling and its target genes Bcl-2 and Bax in the DMBA-induced hamster	12.5 mg/kg, 25 mg/kg, 50 mg/kg	In vivo	[128]
Liver cancer	Gold-Q1 into poly(dl-lactide-*co*-glycolide) nanoparticles	Suppress liver cancer progression via repressing AP-2β/hTERT, impeding caspase/cyto-c pathway, inactivating NF-κB/COX-2 and preventing Akt/ERK1/2 signaling pathways	30 µg/ml, 40 µg/ml, 50 µg/ml	In vitro, in vivo	[68]
	Quercetin	Have hepatoprotective activity versus bile duct ligation caused liver damage through decreasing of Rac1 and NADPH oxidase1 expression	30 mg/kg	In vivo	[129]
	Quercetin	Decrease migration and invasion of HCCLM3 Cells by impeding the expression of p-Akt1, MMP-2, and MMP-9	20 μmol, 40 μmol, 60 μmol	In vitro	[130]
Prostate cancer (PCa)	Quercetin	Represses PCa via attenuating cell survival and frustrating anti-apoptotic pathways	40 μM	In vitro	[69]
	Quercetin	Midkine decreasing lead to increases the effectiveness of Q1 on PCa stem cell survival and migration via PI3K/AKT and MAPK/ERK pathway	29 μM, 35 μM	In vitro	[131]
	Quercetin	Converts the doxorubicin resistance of PCa cells via targeting the c-met/PI3K/AKT pathway	10 μM	In vitro	[132]
Thyroid cancer	Quercetin	Decreases cell proliferation and promoted apoptosis through caspase activation and downregulation of Hsp90 expression	10 μM, 5 μM	In vitro	[59]
	Quercetin	Promotes activator protein1(AP1)activation in FRTL-5 thyroid cells	10 μM	In vitro	[73]
	Hyaluronic acid hydrogel loaded with Q1	Anti-inflammatory action by evaluating IL-4, IL-10, IL-8, IL-1a, and TNFα cellular secretion	2.8 mg/ml, 4.2 mg/ml, 8.4 mg/ml	In vitro	[133]
Hematological malignancies	Quercetin	Enhances the effect of TRAIL-induced apoptosis in KG-1 cells, increased the expression level of DR genes including DR4 and DR5, reducing expression of p65 and c-IAP1, c-IAP2, and XIAP	100 µM	In vitro	[83]
	Quercetin	Provokes apoptosis, cell cycle, and autophagy by decreasing expression of anti-apoptotic proteins, BCL-2, BCL-XL and MCL-1 and increasing expression of BAX, activation of caspase-3, G1 phase cell cycle arrest and inducing conversion of LC3-I to LC3-II	120 mg/kg	In vitro, in vivo	[134]
	Quercetin	Promotes apoptosis and autophagy in primary effusion lymphoma cells via repressing PI3K/AKT/mTOR and STAT3 signaling pathways	50 μM	In vitro	[135]
Lung cancer	Quercetin	An antiproliferative and antimetastatic effect on A549 non-small cell lung cancer cells via the impact on the cytoskeleton	74 μM	In vitro	[136]
	Quercetin	Represses the metastatic capacity of lung cancer via suppressing, Snail-dependent Akt activation and Snail-independent ADAM9 expression pathways	10μM, 50 μM	In vitro, in vivo	[137]
	Quercetin	Promotes apoptosis in the lung cancer via modulation of p53 posttranslational modifications	100 mg/kg	In vivo	[138]
Pancreatic cancer	Quercetin	Induced miR-200b-3p expression and consequently lead to modulate the form of self-renewing divisions in pancreatic cancer	50 μM	In vitro	[70]
	Quercetin	Overexpression of microRNA let-7c and suppress pancreatic cancer progression via activation of Numbl	50 μM	In vivo, in vitro	[95]
	Quercetin	Promoted TRAIL-induced apoptosis through JNK activation-mediated cFLIP turnover	30 μM, 60 μM, 90 μM	In vitro	[96]

## Quercetin as novel therapy for ovarian cancer

Ovarian cancer is one of the most prevalent types of women malignancies and is responsible for the most deaths among the gynecologic cancers. In this cancer, there are some of the risk factors such as age, family history, late menopause, and null parity whiles with the pregnancy and breastfeeding reduce the occurrence risk [95].

The biology of ovarian cancer differs from that of hematogenously metastasizing tumors because ovarian cancer cells primarily disseminate within the peritoneal cavity and are only superficially invasive. Nevertheless, since the rapidly proliferating tumors compress visceral organs and are only temporarily chemosensitive, ovarian carcinoma is a deadly disease [96] Ovarian cancers are separated into type I (low grade) and II (high-grade serous and carcinosarcoma) [97].

In the quest to produce medicines for cancer chemotherapy, Tiwari and colleagues investigated the effectiveness of the combination of drugs operating synergistically. They have studied the effect of Graphene oxide polyvinylpyrrolidone- quercetin -gefitinib (GO-PVP-QSR-GEF) on the ovarian cancer cells (PA-1). They illustrated that quercetin could has a synergic effect on gefitinib anti-cancer property on PA-1 cells, an ovarian cancer originated cell line [98]. In addition, it revealed that quercetin decreased the cell viability of PA-1 cells in a dose and time-dependent procedure. It is worth noticing that they reported 75 μM of quercetin as optimal dose. The molecular mechanism evaluation following the promoting apoptotic effect of quercetin in PA-1 cell showed that quercetin regulates the intrinsic apoptotic pathway that lead to reducing of the anti-apoptotic molecules such as Bcl-2, Bcl-xL while increased the expression level of pro-apoptotic molecules such as caspase-3, caspase-9, cyto-c, Bid, Bad, and Bax [99]. Doxorubicin is a chemotherapeutic agent that used in various cancer including ovarian, thyroid, breast, and lung cancer but a notable unfavorable effect such as cardiotoxicity limits its use [100]. To overcome this limit, scientific used quercetin as a chemosensitizer in combination with doxorubicin. Survey in ovarian cancer xenograft model indicated that quercetin is able to attenuate the doxorubicin-induced cardiotoxicity. Ren et al. investigated the function of quercetin on the proliferation and apoptosis of ovarian cancer cell line SKOV-3 in order to present an experimental foundation for the clinical utilization of quercetin in the therapy of ovarian cancer. Quercetin repressed the proliferation of ovarian cancer SKOV‑3 cells in a time‑ and dose‑dependent manner. Also, quercetin could promote the apoptosis of SKOV-3 cells and decreased the survivin protein expression. Flow cytometry report revealed that quercetin provoked SKOV‑3 cells cycle arrest in the G0/G1 and a notable reduction in the rate of cells at the G2/M phase; moreover, the apoptosis percentage was recognized to boost resulting quercetin therapy [101].

Current evidences about quercetin effects on enhancing the sensitivity of tumor radiotherapy have revealed that quercetin could enhance the effect of radiation-induced cell death. The combination quercetin treatment with X-irradiation increased the DNA damages and created common apoptotic cell death; as well resulted in increasing of Bax level and decreasing of Bcl-2 level in ovarian cancer cell lines (OV2008 and SKOV3) compared to cells presented to quercetin or X-rays alone. In addition, a combination of quercetin treatment with radiation remarkably suppressed the growth of tumors, followed by the induction of p53, CCAAT/enhancer-binding protein homologous protein (CHOP) that is the ER stress biomarker, and γ-H2AX. Conclusively, these outcomes showed that quercetin served as a hopeful radiosensitizer by p53-dependent ER stress signals in human ovarian tumor xenograft model [102].

Quercetin can result in stimulate the ER stress pathway that lead to the cause of cell death and apoptosis [103–105]. Quercetin provoked the ER stress by directly provoking apoptosis via raised levels of GRP78, CHOP that both of them are markers of ER stress and cleaved caspase 4 that has been recognized as a principal performer in ER-induced apoptosis in ovarian cancer cell lines and primary ovarian cancer cells [20, 106]. In order to explore the mechanism of quercetin-induced ER stress and apoptosis, some researchers have focused on the p-STAT3/Bcl-2 axis, which was verified to be able to modulate caspase-cleavage to reduce apoptosis. The outcomes determined that quercetin-induced ER stress is associated with the mitochondrial apoptosis pathway via activation of the p-STAT3/Bcl-2 axis. Unexpectedly, the repression of ER stress could not repeal quercetin-induced apoptosis. Additional functional investigations exhibited that quercetin-induced ER stress could stimulate protective autophagy simultaneously by arousing the p-STAT3/Bcl-2 axis in this manner. Furthermore, 3-MA, an autophagy scavenger, was presented to improve quercetin’s anticancer influences in an ovarian cancer mice xenograft model. Ultimately, this study introduced a unique function of ER stress as a “double edge sword” engaging in quercetin-induced apoptosis in ovarian cancer and might present a new viewpoint to recognize in clinical investigations of biological modifiers that may evade drug resistance in subjects through targeting protective autophagy pathways [20].

Quercetin, because of its little absorption in gastrointestinal tract, enormous first pass metabolism, gastrointestinal instability, low hydrophilicity, and poor solubility, is a challenging pharmacological agent to be delivered [107]. Its oral bioavailability has been illustrated to be less than 17% in animals and less than 2% in human, hence restricting its clinical use utilizing conventional dosage forms [108, 109]. Therefore, an ameliorated quercetin oral formulation is needed with higher efficacy and better bioavailability.

Nanoformulation-based approaches for potential therapeutic molecules delivery have been shown to ameliorate the efficiency of such molecules, which otherwise suffer from particular restrictions [110–112]. Various delivery systems have been demonstrated for quercetin, including microemulsion, nanoparticles, liposomes, and solid lipid nanoparticles for different applications, including in aging and neurodegenerative disease [109, 113–115].

Hydrophobic drugs that are encapsulated into nanoparticles become completely dispersible in water and injectable intravenously. Anticancer drugs which are delivered by biodegradable polymeric nanoparticles, as great candidates for anticancer drug delivery systems [116], are currently used in clinical practice. Poly(3-caprolactone)/poly(ethylene glycol)(PCL/PEG) block copolymers are easy to produce, amphiphilic and biodegradable, indicating hopeful application in drug delivery systems [117]. Recently, utilizing the PCL/PEG nanoparticles for encapsulating drugs to ameliorate the water solubility of hydrophobic drugs has attracted attentions. Gao and colleagues evaluated quercetin nanoformulation effects on the colony formation ability and proliferation of ovarian cells [95]. Encapsulated quercetin into biodegradable monomethoxy poly (ethylene glycol)-poly(ε-caprolactone) (MPEG-PCL) micelles and attempted to yield proof-of-principle for the treatment of ovarian cancer with this quercetin nano-formulation. These quercetin loaded MPEG-PCL (QU/MPEG-PCL) micelles with drug loading of 6.9% had a mean particle size of 36 nm, providing the complete quercetin dispersion in water.

The growth of A2780S ovarian cancer cells was suppressed in vitro by quercetin on a concentration-dependent approach. In addition, intravenously administered QU/MPEG-PCL micelles remarkably inhibited the xenograft A2780S ovarian tumors growth in vivo by suppressing angiogenesis and inducing apoptosis of cancer cells, which is correlated with caspase-3 and -9 activation. In addition, alteration of mitochondrial trans-membrane potential, downregulation of Bcl-2 and MCL-1, and upregulation of Bax were seen, indicating that apoptosis induction effects is via the mitochondrial apoptotic pathways. Anti-proliferative effect of quercetin against A2780S cells was through decreasing phosphorylated Akt as well as phosphorylated p44/42 mitogen-activated protein kinase. Therefore, QU/MPEG-PCL micelles were shown to be novel quercetin nano-formulations with a beneficial application in the treatment of ovarian cancer [95].

In order to show the susceptibility of cisplatin-resistant ovarian cancer cells to quercetin and anticancer properties of this compound, Long et al. conducted in vivo and in vitro studies on the impact of PEGylated liposomal quercetin (Lipo-Que) on cisplatin-resistant (A2780cp) and cisplatin-sensitive (A2780s) human ovarian cancer models [118]. The findings revealed that Lipo-Que induced cell cycle arrest as well as apoptosis, and suppressed cell proliferation in both A2780cp and A2780s cells in vitro. Additionally, anticancer feature of Lipo-Que was assessed in both cisplatin-resistant and cisplatin-sensitive human ovarian tumor xenograft models in nude mice. Compared to normal saline, blank liposomes and free quercetin, Lipo-Que considerably inhibited tumor growth in both models. Moreover, immunofluorescence and immunohistochemistry methods demonstrated that Lipo-Que suppressed tumors proliferation, reduced microvessel density, and induced apoptosis in both A2780cp and A2780s tumor models. Thus, Lipo-Que was proposed to be an efficient formulation for tumor growth inhibition in both cisplatin-resistant and cisplatin-sensitive human ovarian cancers [118].

Teekaraman and his colleagues conducted an in vitro study and illustrated that quercetin, at dose of 50 and 75 μM, is able to induce apoptosis in metastatic ovarian cancer cells leading to inhibit their growth [99]. On the other hand, it was recently reported that co-treatment with quercetin and selenium have synergistic radioprotective and cytoprotective impacts on oxidative stress in endometrial adenocarcinoma cells [119]. Table 2 summarized various studies quercetin in ovarian cancer.

**Table 2:** The therapeutic effects of quercetin on ovarian cancer

Type of quercetin	Mechanism(s) and effect(s)	Dose(s)	Model	Refs.
Graphene oxide polyvinylpyrrolidone-Quercetin-gefitinib (GO-PVP-QSR-GEF)	Cocktailed drug system GO-PVP-QSR-GEF has more cytotoxicity than individual and free drugs toward PA-1 ovarian cancer cells compared to IOSE-364 somatic ovarian epithelial cells	10 mg/l	In vitro	[98]
Quercetin	Represses cell growth and induce apoptosis via reducing Bcl-2, Bcl-xL and increasing the expression level of caspase-3, caspase-9, cyto- c, Bid, Bad, and Bax in PA-1cell line	75 μM	In vitro	[99]
Quercetin	Regulate proliferation, apoptosis, and steroid and peptide hormone secretions in ovarian cells via decreasing PCNA and increasing BAX and in pigs cells T production and also IGF-I secreting reduced but in cattle cells, T releasing increased and at the lower concentration (1 or 10 ng/mL) and a high concentration (100 ng/mL), IGF-I releasing promoted and reduced, respectively	1 ng/mL, 10 ng/mL, 100 ng/mL	In vitro, in vivo	[139]
Micellar(nanostructures) resveratrol (R): Quercetin (Q) (mRQ)	Alleviates the ADR-induced cardiotoxicity	2.11 mg/ml	In vitro, in vivo	[140]
Quercetin	Regulates porcine ovarian granulosa cell roles in vitro via the pathway that may incorporate progesterone, cyclin B1 and p53	0.01 μmol/l, 0.1 μmol/l, 1 μmol/l, 10 μmol/l, 100 μmol/l	In vitro	[141]
Quercetin	Intensify tumor radiosensitivity via p53-mediated ER stress pathways	100 μmol/l	In vitro, in vivo	[102]
Quercetin	Combination treatment of Q1 with xylene shows restricted the effect of xylene on proliferation and IGF-I release, provoked the instigator operation of xylene on apoptosis, and increased the impact of xylene on the release of progesterone but not testosterone	1 μg/ml, 10 μg/ml, 100 μg/ml	In vitro	[20]
Quercetin	Provokes protective autophagy and apoptosis via ER stress by the p-STAT3/Bcl-2 axis in ovarian cancer	40 μM, 80 μM	In vitro, in vivo	[101]
Quercetin	Leads to repression of proliferation and the initiation of apoptosis in ovarian cancer SKOV-3 cells; but, the precise mechanism still needs more investigation	0.12 mg/ml, 0.23 mg/ml, 0.47 mg/ml, 0.94 mg/ml, 1.88 mg/ml, 3.75 mg/ml, 7.5 mg/ml, 15 mg/ml, 30 mg/ml	In vitro	

## Conclusion

It is widely accepted that ovarian cancer is the main cause of deaths associated with gynecology cancer across the world. Utilizing compounds derived from plants has been extensively considered for the treatment of cancer. According to the obtained evidences, quercetin can prevent ovarian cancer through a couple of mechanisms including anti-inflammation, pro-oxidation, anti-proliferation, and cell cycle arrest. Moreover, this natural compound is capable of strengthening the impacts of other chemotherapeutic medications. Further studies should also perform to illustrate its detailed mechanisms of action regarding ovarian cancer. Besides various advantages, utilization of quercetin is associated with different limitations such as very poor bioavailability, poor absorption, rapid metabolism, chemical instability, and rapid systemic elimination. Utilization of quercetin analogs and targeting quercetin by nanotechnology-based approaches may therefore overcome these limitations. Hence, it seems that these platforms can potentially open new horizons in the utilization of quercetin as powerful therapeutic agent alone or in combination with other drugs in the treatment of different types of cancer such as ovarian cancer. So far, there have not been clinical studies to assess the anti-ovarian cancer effects of quercetin. It seems that clinical studies will help us to assess the safety and potency of this drug for the treatment of ovarian cancer.

## Data Availability

Not applicable.

## Abbreviations

COX: Cyclooxygenase
CRP: C-reactive protein
CRC: Colorectal cancer
GC: Gastric cancer
JNK: N-terminal kinase
PBA: Phenyl boronic acid
ROS: Reactive oxygen species
uPA: Urokinase plasminogen activator
